# Removal of Carbamazepine in Aqueous Solution by CoS_2_/Fe^2+^/PMS Process

**DOI:** 10.3390/molecules27144524

**Published:** 2022-07-15

**Authors:** Tingting Wu, Huan Peng, Xiaowei Liu, Ruijin Wu

**Affiliations:** 1 Women’s Hospital, School of Medicine, Zhejiang University, Hangzhou 310006, China; wutt@zju.edu.cn; 2 Zhejiang Key Laboratory of Drinking Water Safety and Distribution Technology, Zhejiang University, Hangzhou 310058, China; huanpeng9@126.com; 3 WISDRI Engineering and Research Incorporation Limited, No.33, Daxueyuan Rd., Wuhan 430070, China; 4 Ocean College, Zhejiang University, Hangzhou 310058, China

**Keywords:** cobalt disulfide, persulfate, carbamazepine, advanced oxidation process

## Abstract

Carbamazepine (CBZ), as a typical pharmaceutical and personal care product (PPCP), cannot be efficiently removed by the conventional drinking water and wastewater treatment process. In this work, the CoS_2_/Fe^2+^/PMS process was applied for efficient elimination of CBZ. The CBZ removal efficiency of CoS_2_/Fe^2+^/PMS was 2.5 times and 23 times higher than that of CoS_2_/PMS and Fe^2+^/PMS, respectively. The intensity of DMPO-HO• and DMPO-${\mathrm{SO}}_{4}^{•-}$ followed the order of Fe^2+^/PMS < CoS_2_/PMS < CoS_2_/Fe^2+^/PMS, also suggesting the CoS_2_/Fe^2+^/PMS process has the highest oxidation activity. The effects of reaction conditions (e.g., CoS_2_ dosage, Fe^2+^ concentration, PMS concentration, initial CBZ concentration, pH, temperature) and water quality parameters (e.g., ${\mathrm{SO}}_{4}^{2-}$, ${\mathrm{NO}}_{3}^{-}$, $H_{2}{\mathrm{PO}}_{4}^{-}$, ${\mathrm{Cl}}^{-}$, ${\mathrm{NH}}_{4}^{+}$, humic acid) on the degradation of CBZ were also studied. Response surface methodology analysis was carried out to obtain the best conditions for the removal of CBZ, which are: Fe^2+^ = 70 µmol/L, PMS = 240 µmol/L, CoS_2_ = 0.59 g/L. The sustainability test demonstrated that the repeated use of CoS_2_ for 8 successive cycles resulted in little function decrease (<10%). These findings suggest that CoS_2_/Fe^2+^/PMS may be a promising method for advanced treatment of tailwater from sewage treatment plant.

## 1. Introduction

Pharmaceuticals and personal care products (PPCPs), which cover a series of chemical substances including various prescription drugs, over-the-counter drugs, cosmetics, and their metabolic transformation products, are a class of emerging organic pollutants that have been widely addressed [1]. There are more than 3000 kinds of drugs in the world that are used in human society. With the development of modern medicine and chemical technology, the types of PPCPs are increasing year by year. However, most of the PPCPs are not fully utilized or absorbed, but finally enter water bodies, including drinking water sources, through sewage discharge, making the water environment a major storage repository for PPCPs. More than 100 types of PPCPs with concentration range from ng/L to μg/L level have been detected in surface water, groundwater, drinking water, and sewage plants in the America, Europe, Asia, and other countries [2,3,4].

Although the concentration of PPCPs detected in the water environment is relatively low, PPCPs have pseudo-persistence, some of them even have bioaccumulation and slow biodegradability in the ecosystem, posing risks to the ecological environment and human health [5]. Considerable evidence suggests that PPCPs remaining in the water environment are affecting environmental organisms and humans in various ways. It can cause gender disorder in environmental organisms, affect the human endocrine system, nervous system, and immune system, and even cause cancer [6,7]. However, current municipal wastewater treatment and drinking water treatment processes cannot effectively remove these pollutants. Therefore, it is still a scientific challenge to develop new approach for PPCPs removal.

The activated persulfate (PS, i.e., peroxomonosulfate (PMS), peroxydisulfate (PDS)) oxidation process based on sulfate radical (${\mathrm{SO}}_{4}^{•-}$) is one of the effective methods to degrade micro-organic pollutants in water, which has received extensive attention in recent years [8]. The activations of PS by heat, ultraviolet, microwave, alkaline, zero-valent metal, transition metal ion/oxide, Fe-based metallic glass, carbon-based material, quinone organics, etc. have been extensively investigated in the past several decades [9,10,11,12,13]. Among these activation methods, activation of PS by transition metal ions such as Fe^2+^, Co^2+^, Mn^2+^, Ni^2+^, etc. [14,15,16] have been widely studied due to the good activation capability, easy accessibility, low energy consumption, and mild temperature requirement. PMS activation by Fe^2+^ (Fe^2+^/PMS) ranks among the options considered to be practical [17,18]. Unfortunately, limitations such as the competition consumption of ${\mathrm{SO}}_{4}^{•-}$ by Fe^2+^, slow regeneration of Fe^2+^, high acid consumption, iron mud production, and low PMS utilization rate, decrease the lure of the Fe^2+^/PMS process. Recently, metal sulfide such as MoS_2_ was reported to be capable of accelerating the Fe^3+^/Fe^2+^ conversion in the Fe^2+^/H_2_O_2_ process [19]. The S atoms on the surface of metal sulfides can capture protons to form H_2_S and expose Mo^4+^ active sites to greatly accelerate Fe^3+^/Fe^2+^ cycling, which could improve H_2_O_2_ decomposition to generate radicals. Metal sulfides may also enhance Fe^2+^/PMS to degrade micro-organic pollutants. Notably, little work has been reported about the depollution efficiency and mechanism of Fe^2+^/PMS in the presence of metal sulfide.

Herein, cobalt disulfide (CoS_2_), as a typical metal sulfide, was used as the aid catalyst of the Fe^2+^/PMS process to degrade PPCPs. Carbamazepine, which is widely used in the treatment of polyuria, arrhythmia, bipolar disorder, and other diseases, was used as a typical PPCP to study the degradation efficiency of CoS_2_-assisted Fe^2+^/PMS process (CoS_2_/Fe^2+^/PMS). The synesthetic mechanism of CoS_2_/PMS and Fe^2+^/PMS, the effects of water quality and operation parameters on CBZ removal efficiency, and response surface methodology (RSM) analysis, as well as the sustainability of the CoS_2_ co-catalyst, were investigated in detail. The purposes of this study are: (1) to offer a new process for CBZ removal and (2) to reveal the mechanism of CoS_2_ for boosting the oxidation activity of Fe^2+^/PMS process.

## 2. Results and Discussion

### 2.1. Degradation Effeciency of CBZ

**Figure 1:**
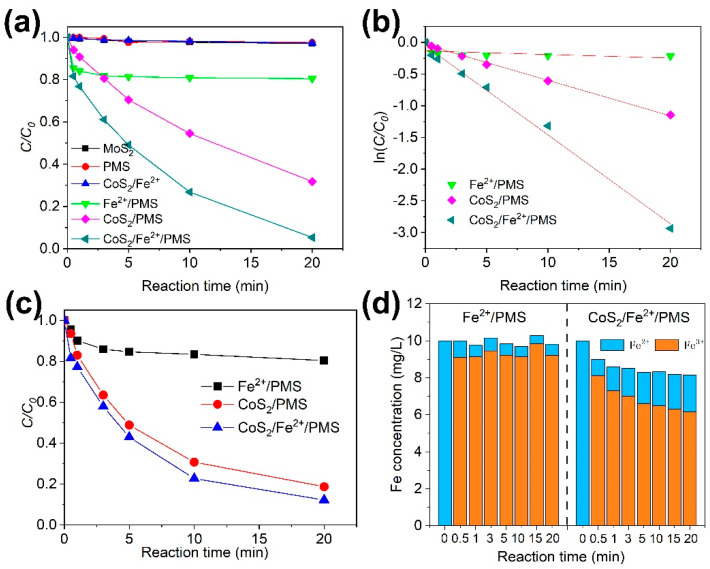
(**a**) The removal efficiency of CBZ by different processes; (**b**) pseudo–first–order kinetic fitting of different processes; (**c**) evolution of PMS concentration in the PMS–based reaction processes; (**d**) evolution of concentrations of Fe^2+^ and Fe^3+^ in the CoS_2_/Fe^2+^/PMS and Fe^2+^/PMS processes. Reaction conditions: T = 25 °C, pH = 3, Fe^2+^ = 17.5 µM, CBZ = 10 mg/L, PMS = 160 µM, CoS_2_ = 0.3 g/L.

The degradation of CBZ by six different processes including CoS_2_, PMS, CoS_2_/Fe^2+^, Fe^2+^/PMS, CoS_2_/PMS, and CoS_2_/Fe^2+^/PMS was studied, and the results are shown in Figure 1a. The removal rates of CBZ after 20 min of reaction were only 2.9% and 2.4%, respectively, by the processes of CoS_2_ or PMS alone, indicating that the adsorption of CBZ on CoS_2_ is very weak, and CBZ can hardly be removed by PMS without the addition of an activator. The CBZ percentage degradation rate of CoS_2_/Fe^2+^/PMS was 94.7%, which was 92%, 75.2%, and 26.5% higher than those of CoS_2_/Fe^2+^, Fe^2+^/PMS, and CoS_2_/PMS, respectively. Further analysis of reaction rate constants (*k*) was conducted to verify whether synesthetic effect between CoS_2_/PMS and Fe^2+^/PMS existed. The degradation curves of PMS-based processes were fitted by the pseudo–first–order kinetic model (Figure 1b), which can be expressed as Equation (1):

ln(*C/C_0_*) = *k*t (1)

where *C* and *C_0_* represent the concentrations of CBZ at the 0 and t time, respectively, *k* is the apparent rate constant. The *k* value of the CoS_2_/Fe^2+^/PMS process was 0.14 min^−1^, which is 2.5 times and 23 times higher than that the processes of CoS_2_/PMS and Fe^2+^/PMS, respectively. This means that the addition of CoS_2_ can significantly improve the degradation efficiency of CBZ by the process of Fe^2+^/PMS. The PMS and Fe^2+^ concentration evolution in the CoS_2_/Fe^2+^/PMS process was measured to evaluate PMS utilization rate and Fe^2+^ regeneration. CoS_2_/Fe^2+^/PMS showed a PMS utilization rate of 87.8%, which is 68.2% and 6.5% higher than those in the processes of Fe^2+^/PMS and CoS_2_/PMS, respectively (Figure 1c). As shown in Figure 1d, the Fe^2+^ was completely oxidized in the first 5 min for Fe^2+^/PMS, leaving most of the remaining PMS unutilized. By contrast, the Fe^2+^ concentration of CoS_2_/Fe^2+^/PMS was slightly increased during the whole reaction process, leading to the full utilization of the PMS.

### 2.2. Effects of Operational Parameters on the Removal of CBZ

**Figure 2:**
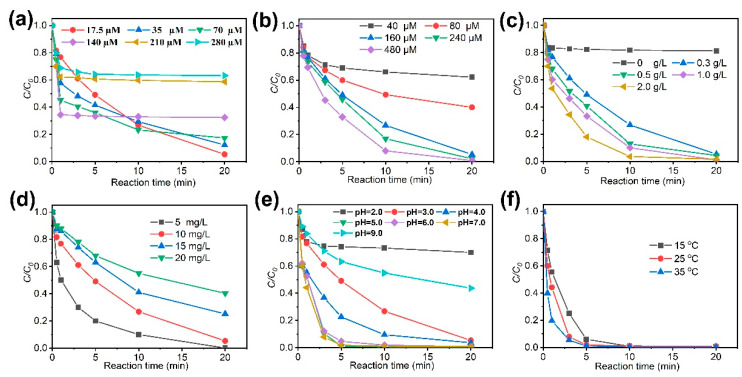
The effects of (**a**) Fe^2+^ concentration, (**b**) PMS concentration, (**c**) CoS_2_ dosage, (**d**) initial CBZ concentration, (**e**) pH, and (**f**) temperature on the removal rates of CBZ by the process of CoS_2_/Fe^2+^/PMS.

In order to optimize the degradation efficiency of the CoS_2_/Fe^2+^/PMS, the operational parameters such as CoS_2_ dosage, Fe^2+^ concentration, PMS concentration, initial CBZ concentration, pH, and temperature were studied. When the Fe^2+^ concentration gradually increased from 17.5 to 70 µM, the degradation efficiency of CBZ gradually increased (Figure 2a). However, when the Fe^2+^ concentration exceeded 140 µM, the degradation efficiency was inhibited. This may result from the competitive consumption of radicals by excessive Fe^2+^ [20]. The CBZ removal efficiency was enhanced with the increase of PMS concentration (Figure 2b) and CoS_2_ dosage (Figure 2c) since ${\mathrm{SO}}_{4}^{•-}$ can be produced by the reaction of PMS and CoS_2_. CBZ at concentration of 5, 10, 15, and 20 mg/L can be degraded by 99.9%, 94.7%, 74.8%, and 59.7% in the CoS_2_/Fe^2+^/PMS process after 20 min (Figure 2d). The gradually decreased CBZ removal rate can be attributed to the decreasing supply of radicals for per molar carbamazepine. The CoS_2_/Fe^2+^/PMS process showed the best CBZ removal efficiency at pH values ranged from 5–7. When the pH was <3 or >9, the removal rate of CBZ was significantly reduced (Figure 2e). This is because high pH value will decrease the amount of soluble Fe^2+^ and accelerate the participation of Fe^3+^, which undoubtedly inhibits CBZ degradation. At low pH, the ${\mathrm{SO}}_{4}^{•-}$ is possibly transformed to other side products as well as scavenging effect of H^+^, which is similar to HO• in Fenton process [21]. Furthermore, the Fe^2+^ forms hydrates such as [Fe(H_2_O)_6_]^2+^, [Fe(H_2_O)_6_]^3+^, [Fe(H_2_O)_5_]^2+^, which is not conducive to activation PMS [22]. Therefore, the CBZ degradation efficiency decreased under pH 2. The degradation rate of CBZ is positively related to the solution temperature ranging from 15 to 35 °C (Figure 2f). This phenomenon can be explained by the fact that high temperature is beneficial to reduce the activation energy of reactions and intensifies the thermal movement of the reaction molecules.

### 2.3. Effects of Water Quality Parameters on the Removal of CBZ

**Figure 3:**
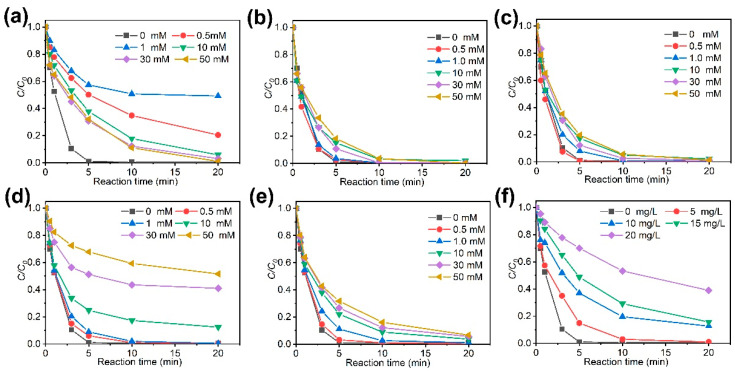
The effects of (**a**) Cl^–^, (**b**) NO_3_^–^, (**c**) SO_4_^2–^, (**d**) H_2_PO_4_^–^, (**e**) NH_4_^+^, and (**f**) HA on the removal rates of CBZ by the process of CoS_2_/Fe^2+^/PMS.

The effects of water quality parameters such as SO_4_^2–^, NO_3_^–^, H_2_PO_4_^–^, Cl^–^, NH_4_^+^, and humic acid (HA) on the degradation of CBZ by the CoS_2_/Fe^2+^/PMS process were also studied (Figure 3). When the concentration of Cl^–^ is low (0.5–1.0 mM), the addition of Cl^–^ showed an inhibitory effect (Figure 3a). This is because ${\mathrm{SO}}_{4}^{•-}$ and HO• can directly oxidize Cl^–^ to produce Cl•, which has lower reactivity toward CBZ than the ${\mathrm{SO}}_{4}^{•-}$ and HO• [23]. With the further increase of Cl^–^ concentration, the accumulation of reactive chlorine species such as Cl• and ${\mathrm{Cl}}_{2}^{•-}$ can compensate the loss of oxidation power. Compared with Cl^–^, the effects of NO_3_^–^ and SO_4_^2–^ on the degradation process were unremarkable (Figure 3b,c). As the concentration of NH_4_^+^ increased from 0 to 50 mmol/L (Figure 3e), the CBZ removal gradually decreased due to the consumption of free radicals to form NO_3_^–^. As to H_2_PO_4_^–^, it showed a weak inhibitory effect on the reaction process at low concentrations of 0–1 mM but decreased the removal rates of CBZ by over 10% at concentrations above 10 mM (Figure 3d). Such inhibition effect originates from the radical scavenging by H_2_PO_4_^–^ and strong complexing between H_2_PO_4_^–^ and Fe^2+^ [24,25]. HA is a typical representative of dissolved organic matter (DOM). The removal rate of CBZ decreased with the increased addition of HA (Figure 3f). This is because HA competes with CBZ, resulting in the decreased degradation rate of CBZ.

### 2.4. Response Surface Methodology Analysis

**Figure 4:**
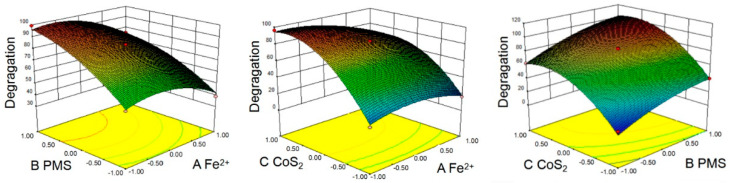
Response surface plots for the effects of any two variables on extraction rate of CBZ degradation rate.

Response surface methodology (RSM) is a method of obtaining a quadratic multiple regression equation through simulation to predict the actual value [26,27]. RSM analyzes the influence of the interaction of various factors on the response value through mathematical and statistical optimization methods and can obtain equations that fit the actual results through limited experimental numerical fitting. To optimize the efficiency of CoS_2_/Fe^2+^/PMS process, three factors, namely A: Fe^2+^ concentration, B: PMS concentration, and C: CoS_2_ dosage, which have a relatively large impact on the CBZ removal rate (screening using Box–Behnken analysis method), were selected. The detailed analysis process is shown in Tables S1–S5 (Supplementary Materials). According to the software simulation, the optimal conditions for the best performance of CoS_2_/Fe^2+^/PMS (99.9% CBZ degradation) are: Fe^2+^ = 70 µM, PMS = 240 µM, and CoS_2_ = 0.59 g/L. Under this condition, the theoretical prediction value of CBZ degradation rate is 100%. The more elliptical shape of the contour line obtained by fitting, the greater the influence of their interaction on the degradation rate, and the rounder the shape of the contour map, the weaker the influence. As shown in Figure 4, the combination of PMS and Fe^2+^ showed the most obvious influence on the degradation process, and the combination of PMS and CoS_2_ ranked the second. The results also indicated that interaction between CoS_2_ and PMS was obvious.

### 2.5. Reaction Mechanism

**Figure 5:**
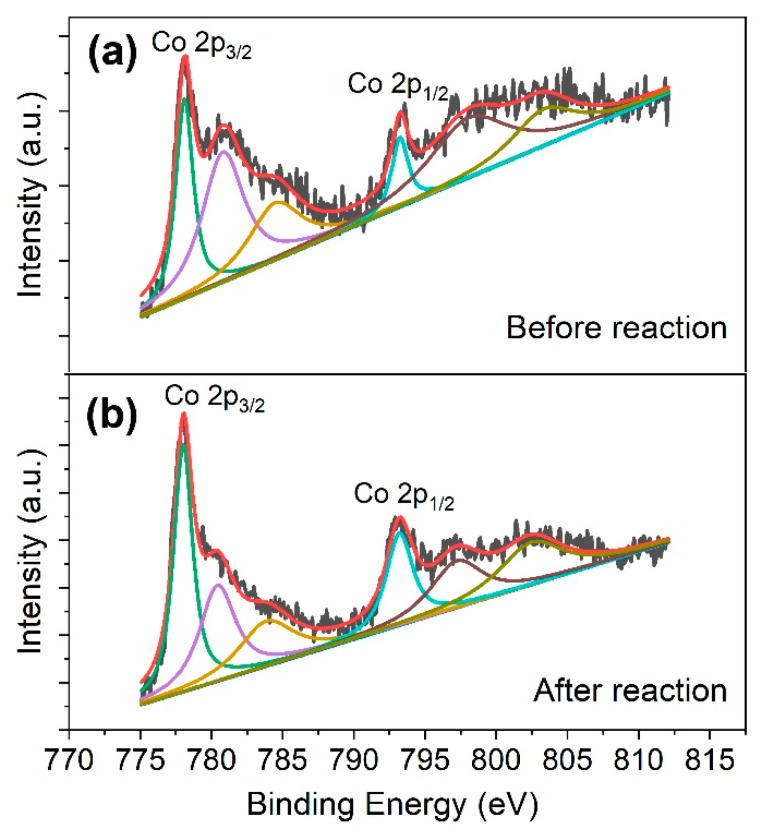
XPS high-resolution spectrum of Co atom before and after reaction in the process of CoS_2_/Fe^2+^/PMS.

It has been reported that the S atoms on the surface of metal sulfides can capture protons to form H_2_S and expose metal active sites to greatly accelerate Fe^3+^/Fe^2+^ cycling, which could improve Fenton decomposition to generate HO• radicals [19]. In order to assess the change of the chemical valence of Co during reaction, the XPS analysis of the CoS_2_ before and after co-catalyst of Fe^2+^/PMS process was performed. Before reaction, two characteristic peaks were identified at 778.2 and 793.5 eV in the Co 2p XPS high-resolution spectrum are related to the spin-orbital splitting of Co 2p_3/2_ and Co 2p_1/2_ respectively (Figure 5a), mainly in the form of Co^3+^ [28,29]. Another two peaks at 780.9 and 797.1 eV are in accordance with Co 2p_3/2_ and Co 2p_1/2_ from Co^2+^ [28,29]. In addition, two peaks at 803.4 and 784.1 eV are ascribed to the satellite peak of Co 2p [28,29,30,31]. When CoS_2_ was dosed in Fe^2+^/PMS system, the characteristic peaks at 780.9 eV (Co^2+^) became smaller and the peak at 778.2 eV (Co^3+^) became larger (Figure 5b), which indicates the Co–S bonds decrease. This phenomenon can be explained by detachment of S on the surface, which will lead to the exposure of Co^2+^ and then facilitates reaction of Co^2+^ with Fe^3+^ to form Co^3+^ and Fe^2+^. In addition, the degradation capability exhibited by CoS_2_/PMS indicates that the exposed Co^2+^ may also be able to activate PMS, which is further confirmed by the EPR determination.

**Figure 6:**
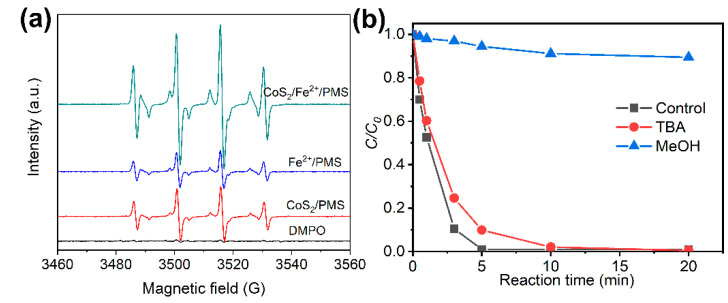
(**a**) EPR spectra of PMS-based reaction processes; (**b**) CBZ degradation by CoS_2_/Fe^2+^/PMS in presence of different quenchers.

To further confirm the addition of the CoS_2_ can boost the oxidation activity of the Fe^2+^/PMS process, the EPR spectra of DMPO-${\mathrm{SO}}_{4}^{•-}$ and DMPO-HO• in the PMS-based reaction processes including Fe^2+^/PMS, CoS_2_/PMS and CoS_2_/Fe^2+^/PMS were detected using an EPR spin trapping technique with the trapping agent DMPO. The typical signal peaks for ${\mathrm{SO}}_{4}^{•-}$ (six-line spins with intensity ratio of 1:1:1:1:1:1, α_N_ = 13.51G, α_β-H_ = 9.93G, α_γ-H1_ = 1.34, α_γ-H2_ = 0.88) and HO• (four-line spins with intensity ratio of 1:2:2:1, α_N_ = α_β-H_ = 14.9 G) were monitored in all of the PMS-based reaction processes (Figure 6a). The intensity of DMPO-HO• and DMPO-${\mathrm{SO}}_{4}^{•-}$ in the PMS-based reaction processes followed the order of Fe^2+^/PMS < CoS_2_/PMS < CoS_2_/Fe^2+^/PMS, which was consistent with the law of their degradation efficiency (Figure 1a). It should be pointed out that the DMPO-HO• peak strength was significantly higher than that of DMPO-${\mathrm{SO}}_{4}^{•-}$. This is because ${\mathrm{SO}}_{4}^{•-}$ can quickly transform into HO• when it is produced in water solution [32] and the signal intensity of DMPO-${\mathrm{SO}}_{4}^{•-}$ adduct spin was inherently much weaker than that of DMPO-HO• adduct spin. In order to identify whether HO• or ${\mathrm{SO}}_{4}^{•-}$ are the main species contributing to CBZ degradation in the CoS_2_/Fe^2+^/PMS system, competitive experiments with different quencher addition were conducted. MeOH and TBA were used for quenching the ${\mathrm{SO}}_{4}^{•-}$ and HO•, respectively. As shown in Figure 6b, the degradation efficiency was significantly reduced with the addition of MeOH (quencher for ${\mathrm{SO}}_{4}^{•-}$ and HO•) but slightly decreased with the addition of TBA (quencher for ${\mathrm{SO}}_{4}^{•-}$ and HO•), indicating that the ${\mathrm{SO}}_{4}^{•-}$ was the main active substance in the process of CoS_2_/Fe^2+^/PMS. Contributions of HO• and ${\mathrm{SO}}_{4}^{•-}$ are 10% and 85%, respectively.

### 2.6. Sustainability of CoS_2_

**Figure 7:**
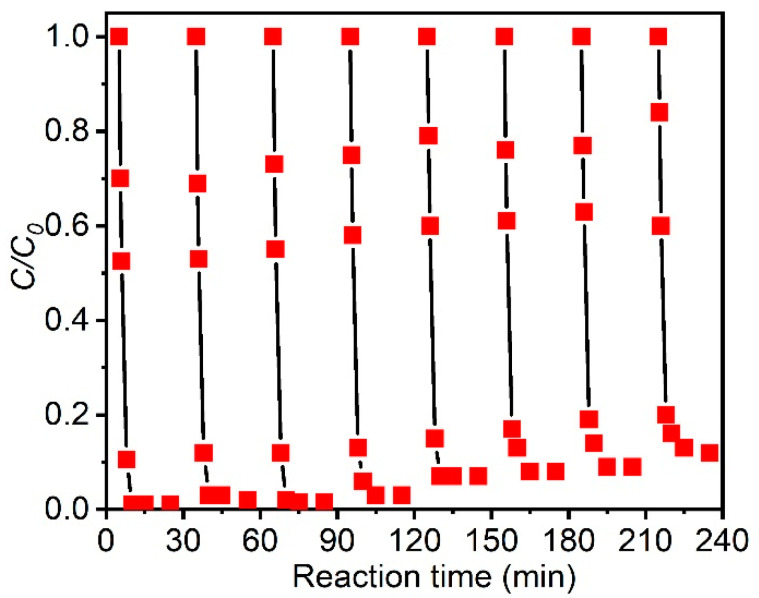
Eight cycles of the degradation of CBZ by the CoS_2_/Fe^2+^/PMS process.

The cycle life and stability of the catalyst are important factors to measure the performance of the catalyst. After 8 cycles, the CBZ removal rate at reaction time of 20 min was still more than 90% by the CoS_2_/Fe^2+^/PMS process (Figure 7), indicating that CoS_2_ has good stability. Moreover, the dissolved Co^2+^ concentration after 8 cycles was only 0.8 mg/L, suggesting the good stability of the CoS_2_. In order to further confirm the stability of CoS_2_, the crystal structure and morphology of CoS_2_ before and after the reaction were analyzed by SEM, TEM, and XRD. After 8 cycles of use, the morphology of CoS_2_ did not change significantly (Figures S1 and S2, Supplementary Materials). The position and intensity of the XRD diffraction peaks did not change, indicating no crystal phase changing during reaction. The SEM, TEM, and XRD results also suggest the good chemical stability of the CoS_2_.

## 3. Materials and Methods

### 3.1. Materials

Cobalt disulfide particles, tert-butyl alcohol (TBA, ≥99%), and potassium peroxymonosulfate (KHSO_5_·0.5KHSO_4_·0.5K_2_SO_4_, ≥47%) were purchased from Shanghai Aladdin Reagent Co., Ltd. (Shanghai, China) Carbamazepine (C_15_H_12_N_2_O, ≥99.0%) was purchased from Tianjin Sinos Opto Technology Co., Ltd., address: Room 2001-14, No. 8 Gaoying Road, Beizhakou Demonstration Town, Jinnan District, Tianjin, China. Ferrous sulfate heptahydrate (FeSO_4_·7H_2_O, >99.0%), sulfuric acid (H_2_SO_4_, ≥99%), sodium hydroxide (NaOH, ≥99%), sodium sulfate (Na_2_SO_4_, ≥99%), sodium nitrate (NaNO_3_, ≥99%), sodium chloride (NaCl, ≥99%), potassium dihydrogen phosphate (KH_2_PO_4_, ≥99%), and ammonium chloride (NH_4_Cl, ≥99%) were purchased from Sinopharm Chemical Reagent Co., Shanghai, China. 5,5-dimethyl-1-pyrroline-Noxide (DMPO, >99%) was obtained from Dojin Chemical Research Institute Co., Ltd., Kumamoto, Japan.

### 3.2. Experimental Procedures

First, 200 mL of CBZ solution with concentrations ranging from 5 to 20 mg/L were added into a 250 mL glass beaker. Then, CoS_2_ suspension with concentrations ranging from 0.3 to 2.0 g/L and Fe^2+^ solutions with concentrations ranging from 17.5 to 280 µmol/L were added into the above CBZ solution under ultrasonication for 30 s. Subsequently, the solution pH was adjusted at the range of 2–9 by NaOH and/or H_2_SO_4_ solution. Finally, the PMS solution with concentrations ranging from 40 to 480 µmol/L were injected into the mixture to start the reaction. Water samples were taken at different time intervals with addition of methanol to quench the reaction. The samples were centrifuged at 10,000 r/min for 5 min to obtain the clean solutions for determination of CBZ concentrations.

### 3.3. Analysis Methods

The CBZ concentrations were determined by using a High-Performance Liquid Chromatography (HPLC) device equipped with a reversed-phase ZORBAX Eclipse XDB-C18 column (4.6 mm × 150 mm, 5 μm) at a UV wavelength of 245 nm. The mobile phase was set at 60/40 water/methanol. The flow rate was set at 1.0 mL/min with an injection volume of 20 µL. The concentration of dissolved Fe^2+^, total Fe ions, and dissolved Co^2+^ were detected by using the colorimetric method and Inductively Coupled Plasma Mass Spectrometry (ICP-MS, PerkinElmer NexION 350Q, Waltham, MA, USA). The crystalline phase and morphologies of the CoS_2_ particles before and after reaction were monitored by an Rigaku X-ray diffractometer (XRD) and an FEI FEG650 field-emission scanning electron microscope (SEM), respectively. Radicals (HO• and ${\mathrm{SO}}_{4}^{•-}$) were determined by a Bruker A300 Electron paramagnetic resonance (EPR) Spectrometer. The EPR spectrometer settings in the spin trapping experiments were as follows: modulation amplitude, 0.1 mT; center field, 351.194 mT; sweep width, 10.00 mT; sweep time, 41 s; microwave power, 2.25 mW; microwave frequency, 9.858 GHz; and receiver gain, 1.42 × 10^4^. X-ray photoelectron spectroscopy (XPS) was used to observe Co valence change.

The PMS concentration was measured by the iodometric method, which was divided into several steps: Step 1: prepare a mixed solution of potassium iodide and sodium bicarbonate with concentration of 100 g/L and 5 g/L, respectively. Step 2: 5 mL of the above mixed solution was added in a 10 mL colorimetric tube, then 0.5 mL of the PMS solution with concentrations of 0.00, 0.01, 0.02, 0.04, 0.06, 0.08, 0.10 mmol/L were added. Step 3: the above solution was shaken vigorously to mix evenly and placed for 20 min to develop color. The absorbance of the colored solution was measured at a wavelength of 352 nm via a UV spectrophotometer. Finally, a standard curve of PMS concentration and absorbance intensity can be obtained (Figure S3, Supplementary Materials).

### 3.4. RSM Experimental Design and Results

The RSM experimental was designed with three factors and three levels. Fe^2+^ concentration (A), PMS concentration (B), and CoS_2_ dosage (C) were selected as the three factors. The three levels in this experiment refer to the low, medium, and high concentrations of the selected factors, which can be represented by −1, 0, +1 respectively. The conversion equation between the code and the actual value is shown in Equation (2):

(2) $$N_{i}=\frac{X_{i}-X_{0}}{\Delta X}$$

Among them, *N_i_* is the variable code value, *X*_0_ is the concentration of the independent variable at the center point of the experiment, and Δ*X* is the change step length of the concentration. The removal rate of CBZ (%) is taken as the response value *Y*. The experimental data were fitted and analyzed using the design software Design Expert. The experimental design scheme shown in Table S1, Supplementary Materials. There are 17 groups of experiments. The experimental scheme is randomly generated and given according to the software.

The experimental data were fitted by Design Expert, and a model including multiple independent variables and response values was obtained. The relationship can be described by Equation (3):

(3) $$Y=\beta_{0}+\overset{k}{\underset{i=1}{\sum }}\beta_{i}x_{i}+\overset{k}{\underset{i=1}{\sum }}\beta_{ii}x_{i}^{2}+\overset{j-1}{\underset{i=1}{\sum }}\overset{k}{\underset{j=1}{\sum }}\beta_{ij}x_{i}x_{j}$$

Equation (4) can be obtained by fitting the data in Table S2, Supplementary Materials.

(4) $$Y_{1}=83.30-8.78\times \mathrm{Fe}+18.34\times \mathrm{PMS}+29.44\times \mathrm{Co}S_{2}-2.33\times \mathrm{Fe}\times \mathrm{PMS}-\;6.28\times \mathrm{Fe}\times \mathrm{Co}S_{2}+1.45\times \mathrm{PMS}\times \mathrm{Co}S_{2}-9.68\times \mathrm{Fe}\times \mathrm{Fe}-7.00\times \mathrm{PMS}\times \;\mathrm{PMS}-23.60\times \mathrm{Co}S_{2}\times \mathrm{Co}S_{2}$$

The analysis of variance of the regression model is shown in Table S3, Supplementary Materials. The F value is 110.66 and *p* value is <0.0001, which means that the *Y_1_* model is extremely significant.

Figure S4 represents the residual probability distribution diagrams of the model predicted value and the actual value. The residual probability of the actual value and the predicted value are all distributed on a straight line, and the actual value and the predicted value are not much different. By comparing the value of F, it can be seen that the factors affecting the reaction process from large to small are: C(CoS_2_) > B(PMS) > A(Fe^2+^).

The simulation model is relatively complex and includes some factors that do not have a significant impact on the experimental results. An overly complex model may cause partial distortion of the model. Therefore, it is necessary to make simple corrections to the model and eliminate the insignificant factors to obtain a better simulation model. A new simulation model (*Y_2_*, Equation (5)) was obtained by fitting the data in Table S4 with two insignificant items (*p* > 0.05) deleted. The analysis of variance of the regression model is shown in Table S5.

(5) $$Y_{2}=83.30-8.78\times \mathrm{Fe}+18.34\times \mathrm{PMS}+29.44\times \mathrm{Co}S_{2}-6.27\times \mathrm{Fe}\times \mathrm{Co}S_{2}\;-9.68\times \mathrm{Fe}\times \mathrm{Fe}-7.00\times \mathrm{PMS}\times \mathrm{PMS}-23.60\times \mathrm{Co}S_{2}\times \mathrm{Co}S_{2}$$

In order to explore the influence of each factor pairwise interaction on CBZ removal rate, Design Expert software was used to draw contour plots and response surface plots of the AB, BC, and AC terms in the regression equation. The more elliptical shape of the contour line obtained by fitting, the greater the influence of their interaction on the degradation rate, and the rounder the shape of the contour map, the weaker the influence. As shown in Figure 4, the interaction between PMS and CoS_2_ concentration was obvious.

## 4. Conclusions

In conclusion, we have demonstrated that carbamazepine (CBZ) can be efficiently removed by the CoS_2_/Fe^2+^/PMS process. The apparent degradation rate constant of CBZ was 0.14 min^−1^ for CoS_2_/Fe^2+^/PMS, which was 2.5 times and 23 times higher than that for CoS_2_/PMS and Fe^2+^/PMS, respectively. The HO• and ${\mathrm{SO}}_{4}^{•-}$ were the two main oxidation species in the reaction processes, which followed the order of Fe^2+^/PMS < CoS_2_/PMS < CoS_2_/Fe^2+^/PMS. The enhanced removal efficiency was due to the reduction of Fe^3+^ by the exposed Co^2+^ on the CoS_2_ particles. We believe the high catalytic oxidation activity, low catalyst dosage, and good stability make the CoS_2_/Fe^2+^/PMS process potential application for CBZ wastewater treatment.

## Data Availability

Not applicable.

## Supplementary Materials

The following supporting information can be downloaded at: https://www.mdpi.com/article/10.3390/molecules27144524/s1, Figure S1: TEM of CoS_2_ (a) before and (b) after 8-cycle reaction; SEM of CoS_2_ (c) before and (d) after 8-cycle reaction. Figure S2: XRD patterns of CoS_2_ before and after 8-cycle reaction. Figure S3: Standard curve for PMS concentration. Figure S4: (a) The residual probability distribution of predicted and actual values; (b) Comparison of actual and predicted values. Table S1: Response surface experimental design. Table S2: Experimental design and results. Table S3: Model analysis of variance. Table S4: Analysis of variance of the regression model *Y_1_*. Table S5: Analysis of variance of the regression model *Y_2_*.

## Sample Availability

Samples of the compounds are not available from the authors.
