# Supplementary material for: Removal of Carbamazepine in Aqueous Solution by CoS2/Fe2+/PMS Process
Source: Molecules. 2022 Jul 15;27(14):4524. doi: 10.3390/molecules27144524 (PMC9323623; doi:10.3390/molecules27144524)
Supplement: Supplementary file 1 [file molecules-27-04524-s001.zip › molecules-1802324-supplementary.pdf]

Supplementary Materials for

Removal of carbamazepine in aqueous solution by CoS_2/Fe^{2+}/PMS process

Tingting Wu[1], Huan Peng[2,3], Xiaowei Liu[2,4,*], Ruijin Wu[1,*]

[1] Women's Hospital, School of Medicine, Zhejiang University, Hangzhou 310006, China.

[2] Zhejiang Key Laboratory of Drinking Water Safety and Distribution Technology, Zhejiang University, Hangzhou 310058, China.

[3] WISDRI Engineering and Research Incorporation Limited. No.33, Daxueyuan Rd., Wuhan 430070, China.

[4] Ocean College, Zhejiang University, Hangzhou 310058, China.

*Corresponding author.

Email address: liuxiaowei@zju.edu.cn (X.L.); wurj@zju.edu.cn (R.W.)

Number of pages: 6

Number of figures: 4

Number of tables: 5

Table S1 Response surface experimental design

Impact factor	unit	code	Impact factor		
			-1	0	1
Fe^{2+}concentrtion	μmol/L	A	17.5	70	122.5
PMS concentration	μmol/L	B	80	160	240
CoS_2 dosage	g/L	C	0.01	0.3	0.59

Table S2 Experimental design and results

Number	A($C_{Fe^{2+}}$)	B(C_{PMS})	C(C_{CoS_2})	Y(Degragation)
1	-1	-1	0	53.3
2	-1	0	-1	19.8
3	0	1	-1	40.3
4	0	-1	-1	11.1
5	0	0	0	83.2
6	1	1	0	75.3
7	1	-1	0	38.7
8	0	1	1	97.2
9	-1	1	0	99.2
10	0	-1	1	62.2
11	0	0	0	82.9
12	1	0	1	67.7
13	1	0	-1	16.5
14	0	0	0	83.6
15	0	0	0	83.7
16	0	0	0	83.5
17	-1	0	1	96.2

Table S3 Model analysis of variance

F	P	R^2	$AdjR^2$	Ap	CV
110.66	<0.0001	0.993	0.984	33.706	5.74

Table S4 Analysis of variance of the regression model Y1

Items	square sum	Freedom degree	mean square	F	P
Model	13606.11	9	1511.79	110.66	< 0.0001
A	616	1	616	45.09	0.0003
B	2690.11	1	2690.11	196.91	< 0.0001
C	6932.53	1	6932.53	507.44	< 0.0001
AB	21.62	1	21.62	1.58	0.2487
AC	157.5	1	157.5	11.53	0.0115
BC	8.41	1	8.41	0.62	0.4584
A^2	394.13	1	394.13	28.85	0.001
B^2	206.32	1	206.32	15.1	0.006
C^2	2345.09	1	2345.09	171.65	< 0.0001

Table S5 Analysis of variance of the regression model Y2

Items	square sum	Freedom degree	mean square	F	P
Model	13576.08	7	1939.44	138.90	< 0.0001
A	616.00	1	616	44.12	< 0.0001
B	2690.11	1	2690.11	192.66	< 0.0001
C	6932.53	1	6932.53	496.5	< 0.0001
BC	157.50	1	157.5	11.28	0.0084
A^2	394.13	1	394.13	28.23	0.0005
B^2	206.32	1	206.32	14.78	0.0039
C^2	2345.09	1	2345.09	167.95	< 0.0001

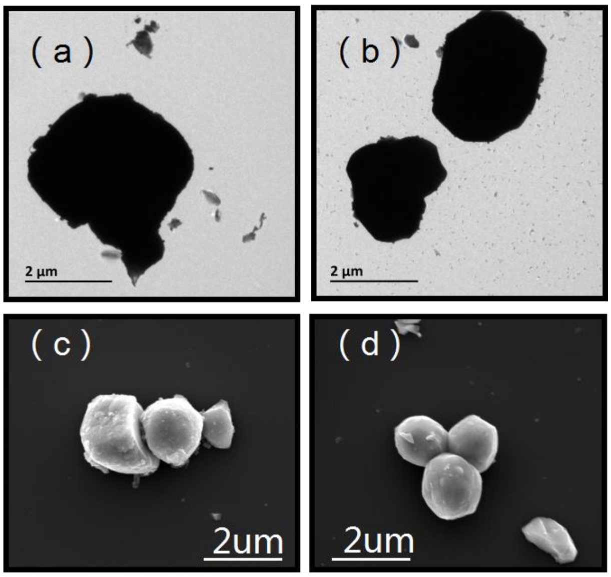

Figure S1. TEM of CoS_2 (a) before and (b) after 8-cycle reaction; SEM of CoS_2 (c) before and (d) after 8-cycle reaction.

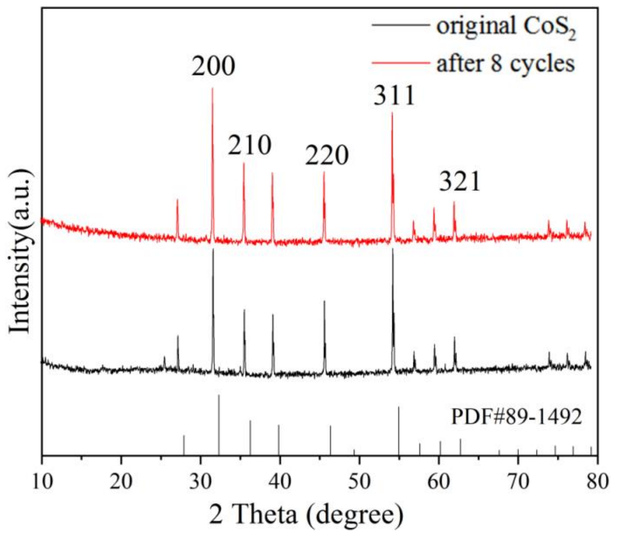

Figure S2. XRD patterns of CoS_2 before and after 8-cycle reaction.

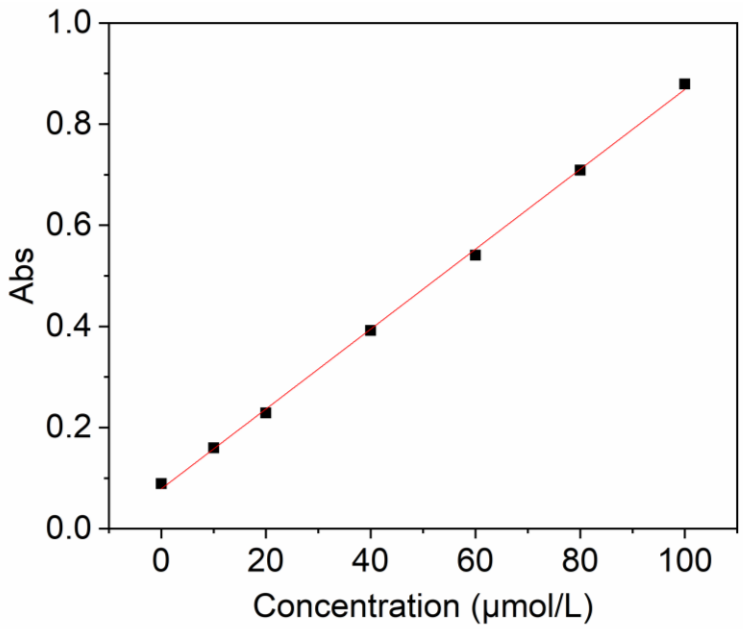

Figure S3. Standard curve for PMS concentration.

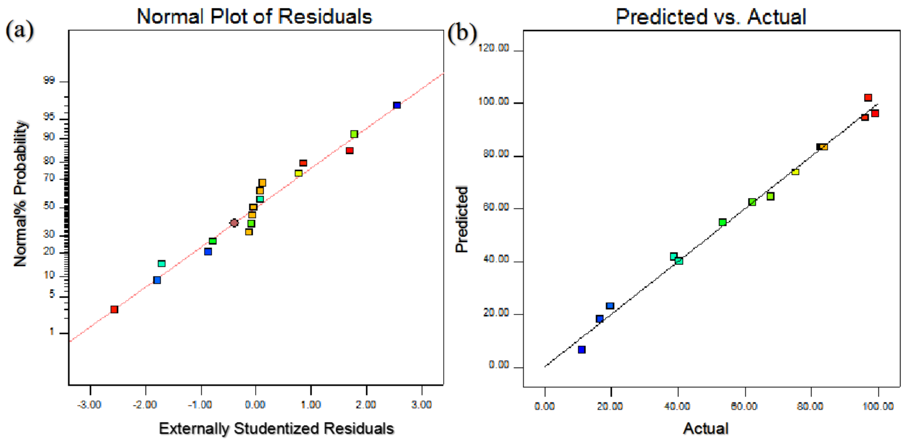

Figure S4. (a) The residual probability distribution of predicted and actual values; (b) Comparison of actual and predicted values.
